# BMC Structural Biology reviewer acknowledgement 2015

**DOI:** 10.1186/s12900-016-0054-8

**Published:** 2016-02-09

**Authors:** Julia Simundza

**Affiliations:** BioMed Central, 233 Spring Street, New York, NY 10013-1578 USA

## Abstract

The editors of *BMC Structural Biology* would like to thank all our reviewers who have contributed to the journal in Volume 15 (2015).

**Jean Armengaud**

France

**Pascal Arnoux**

France

**Oluwatoyin Asojo**

USA

**Faizul Azam**

India

**Lin Bai**

USA

**Andrea Bernini**

Italy

**Yoshitaka Bessho**

Japan

**Ramachandra M Bhaskara**

Germany

**Amadeo Biter**

USA

**Michal Brylinski**

USA

**Susan Buchanan**

USA

**David Burke**

UK

**Weiguo Cao**

USA

**Berenice Carrillo**

USA

**Rita Casadio**

Italy

**Matthew Champion**

USA

**Chi-Cheng Chiu**

Taiwan

**Stefano Ciurli**

Italy

**Nelson Da Silveira**

Brazil

**Peter Davies**

Canada

**Jamaine Davis**

USA

**Bernard Delmas**

France

**Xavier Deupi**

Switzerland

**Giray Enkavi**

Finland

**Sebnem Eşsiz Gökhan**

Turkey

**Federico Fogolari**

Italy

**Samuel Gerritz**

USA

**David Gloriam**

Denmark

**Xinqigong Gong**

China

**Thomas Grant**

USA

**Ulrich Hansmann**

USA

**William Heller**

USA

**Hirotsugu Hiramatsu**

USA

**Alvin Holder**

USA

**Frédéric Iseni**

France

**Andriy Kryshtafovych**

USA

**Andreas Kukol**

UK

**Takashi Kumasaka**

Japan

**Igor Kuznetsov**

USA

**Nicole Laronde**

USA

**Vanessa Leone**

USA

**Oded Lewinson**

Israel

**Zhijun Li**

USA

**Christian Linke-Winnebeck**

Germany

**George Lountos**

USA

**Daniel Macqueen**

UK

**Arumugam Madeswaran**

India

**Megan Maher**

Australia

**Michael Massiah**

USA

**Liam James Mcguffin**

UK

**Erika Merschrod**

Canada

**Elin Moe**

Norway

**Mathur Ramabhadrashastry Narasimha Murthy**

India

**Hartmut Niemann**

Germany

**Hyun Ho Park**

Republic Of Korea

**Olivier Pietrement**

France

**Erik Procko**

USA

**Zihe Rao**

China

**Juan Reguera**

France

**Scott Reiling**

USA

**Daniel Roche**

France

**Markus Rudolph**

Switzerland

**Armin Ruf**

USA

**Gerhard Schenk**

Australia

**Yaming Shao**

USA

**Hiroyuki Sorimachi**

Japan

**Shaun Stauffer**

USA

**Eric Sundberg**

USA

**Mayuko Takeda-Shitaka**

Japan

**Dror Tobi**

Israel

**Thom Vreven**

USA

**Qingguo Wang**

USA

**Wenqing Xu**

USA

**Hye-Jeong Yeo**

USA

**Masafumi Yohda**

Japan

**Hongjun Yu**

USA

